# Supplementary material for: Elevating density functional theory to chemical accuracy for water simulations through a density-corrected many-body formalism
Source: Nat Commun. 2021 Nov 4;12:6359. doi: 10.1038/s41467-021-26618-9 (PMC8569147; doi:10.1038/s41467-021-26618-9)
Supplement: Supplementary file 1 — Supplementary Information [file 41467_2021_26618_MOESM1_ESM.pdf]

Supplementary Information

Elevating Density Functional Theory to Chemical Accuracy for Water Simulations through a Density-Corrected Many-Body Formalism

S. Dasgupta et al.

Supplementary Note 1

Input for a single point energy calculation of H_2O with SCAN(DC)/aug-cc-pVQZ in Q-Chem

```
$molecule
0 1
O      0.00000    -0.06580     0.00000
H      0.00000     0.52218     0.75754
H      0.00000     0.52218    -0.75754
$end

$rem
method hf
jobtype            sp
scf_convergence    11
thresh             14
basis              aug-cc-pVQZ
sym_ignore         true
no_reorient        true
$end
@@@
$molecule
read
$end

$rem
max_scf_cycles 1
```

```
method scan
jobtype         sp
scf_guess       read
thresh          14
basis           aug-cc-pVQZ
sym_ignore      true
no_reorient     true
$end
```

Supplementary Figures

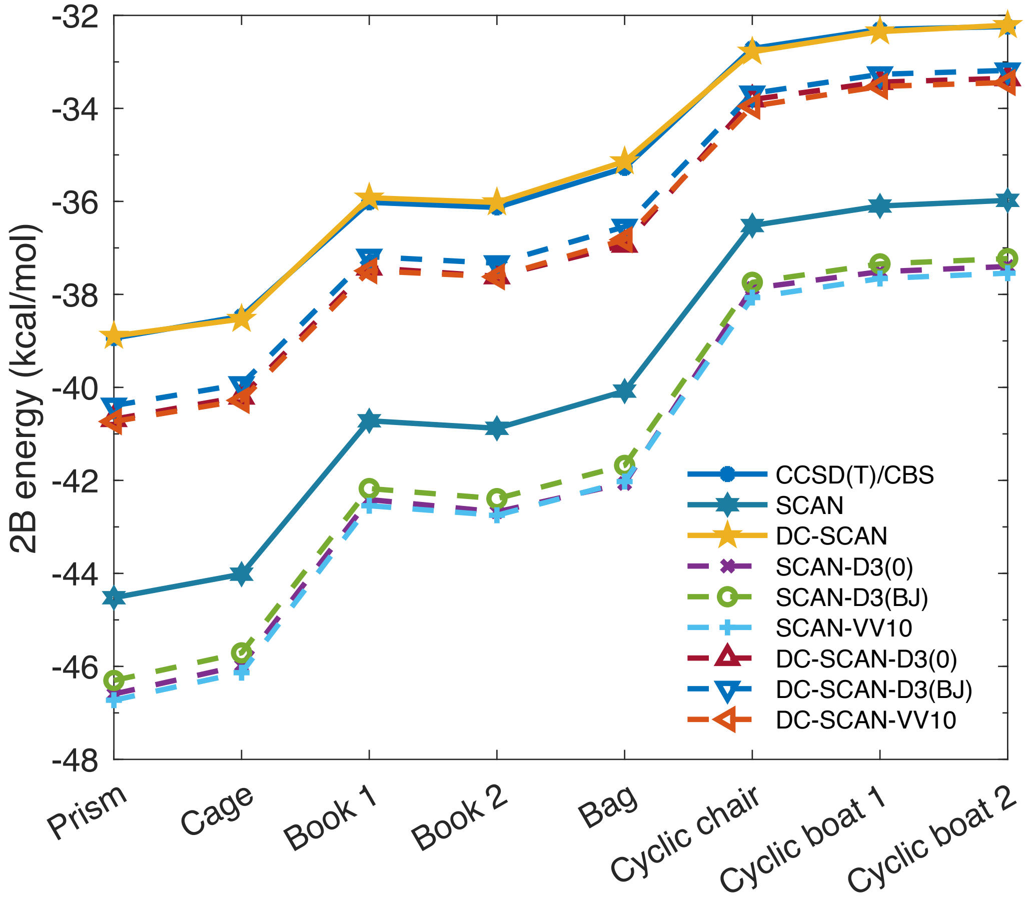

Supplementary Figure 1: **Effects of various dispersion corrections on 2B energies.** 2-body energies calculated for the first eight low-energy isomers of the water hexamer using SCAN, DC-SCAN, SCAN-D3(0), SCAN-D3(BJ), SCAN-VV10, DC-SCAN-D3(0), DC-SCAN-D3(BJ), and DC-SCAN-VV10, along with the corresponding CCSD(T)/CBS reference values from ref. 1. All the dispersion parameters have been taken from ref. 2

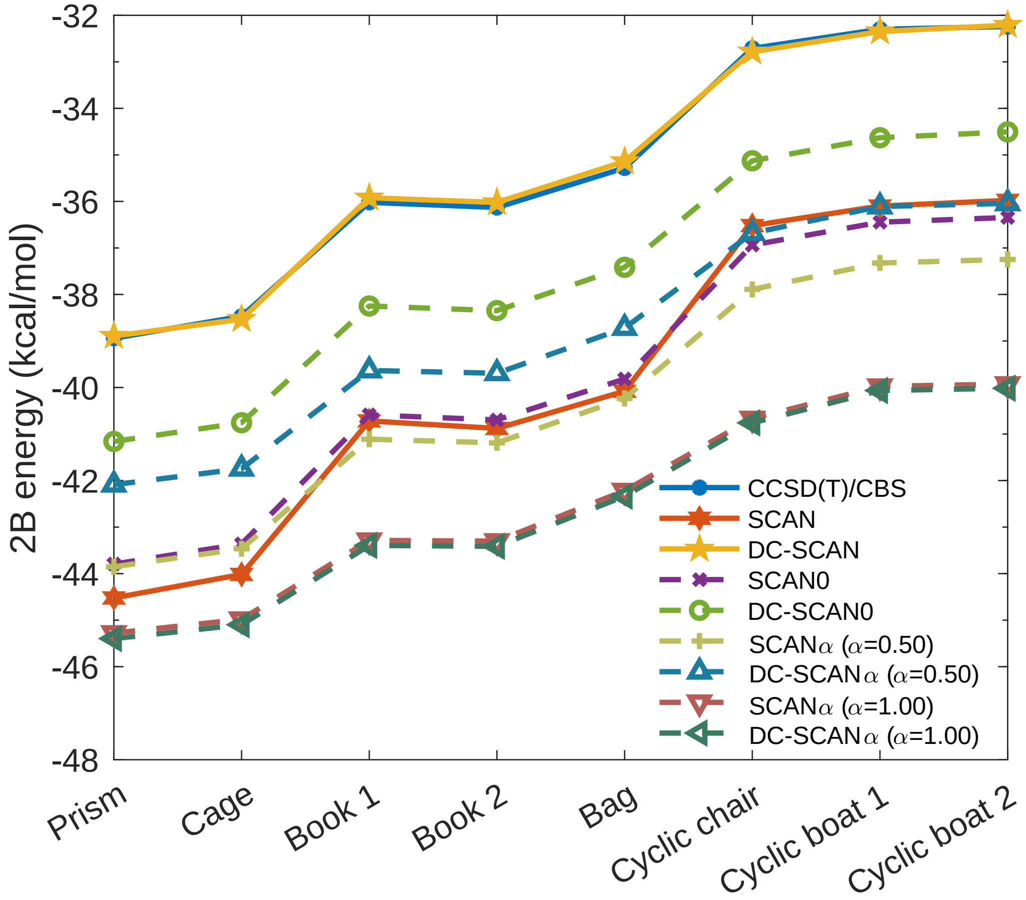

Supplementary Figure 2: **2B energies calculated with SCANα and DC-SCANα.** 2-body energies calculated for the first eight low-energy isomers of the water hexamer using SCAN, DC-SCAN, SCAN0, DC-SCAN0, SCANα (α=0.50), DC-SCANα (α=0.50), SCANα (α=1.00), and DC-SCANα (α=1.00), along with the corresponding CCSD(T)/CBS reference values from ref. 1.

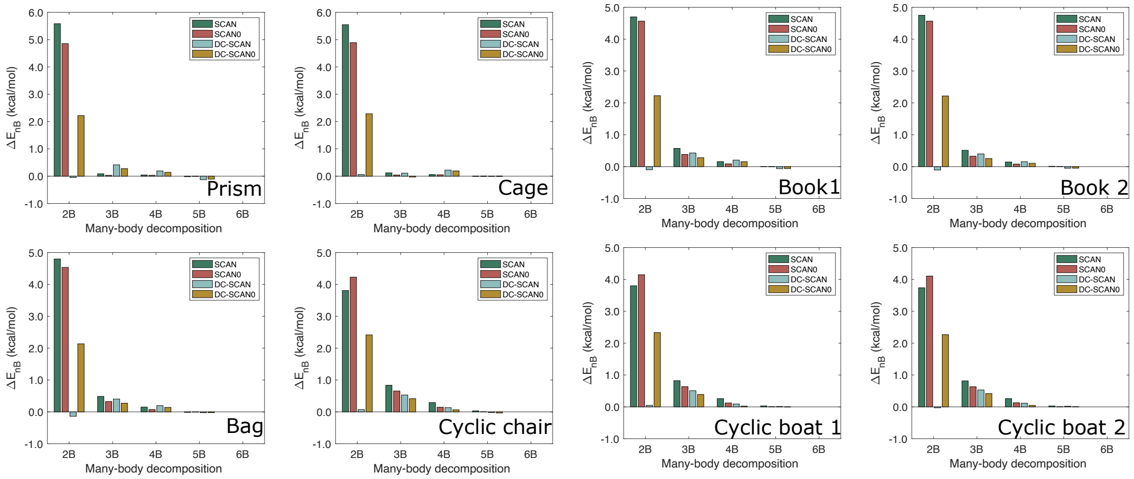

Supplementary Figure 3: **Errors in many-body energies.** Errors in nB energies (with $n = 2-6$) associated with the self-consistent SCAN and SCAN0 functionals, and the corresponding density-corrected DC-SCAN and DC-SCAN0 functionals relative to the CCSD(T)/CBS reference values of ref. 1 calculated for the first eight low-energy isomers of the water hexamer. Errors are defined as $\Delta E^{n\mathrm{B}} = E^{n\mathrm{B}}_{\mathrm{CCSD(T)}} - E^{n\mathrm{B}}_{\mathrm{DFT}}$, where $E^{n\mathrm{B}}_{\mathrm{DFT}}$ and $E^{n\mathrm{B}}_{\mathrm{CCSD(T)}}$ are the nB energies calculated with the self-consistent and density-corrected functionals and at the CCSD(T)/CBS level of theory, respectively.

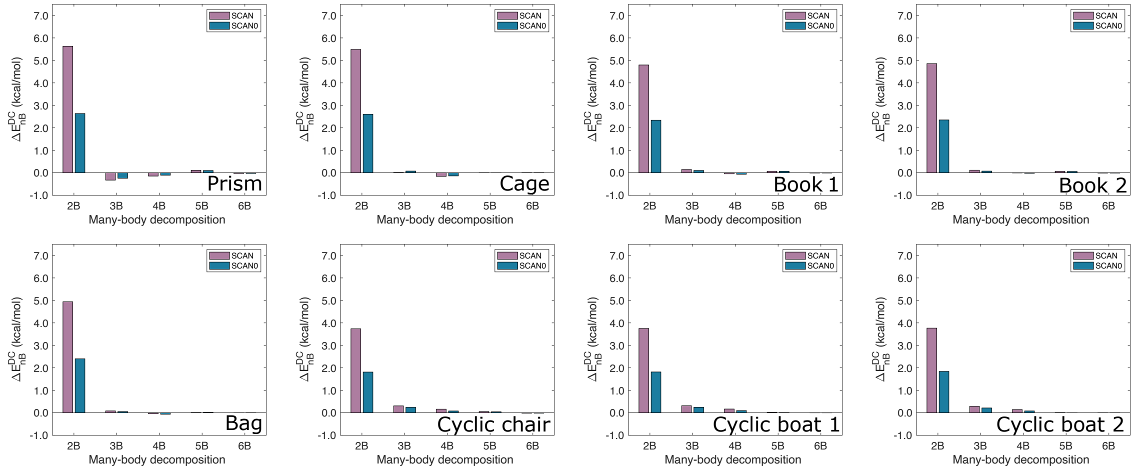

Supplementary Figure 4: **Differences between DC-DFT and SC-DFT many-body energies.** Errors in nB energies (with $n = 2 - 6$) associated with the self-consistent SCAN and SCAN0 functionals relative to the values calculated with the corresponding density-corrected DC-SCAN and DC-SCAN0 functionals for the first eight low-energy isomers of the water hexamer. Errors are defined as $\Delta E^{n\mathrm{B}}_{\mathrm{DC}} = E^{n\mathrm{B}}_{\mathrm{DC}} - E^{n\mathrm{B}}_{\mathrm{SC}}$, where $E^{n\mathrm{B}}_{\mathrm{DC}}$ and $E^{n\mathrm{B}}_{\mathrm{SC}}$ are the nB energies calculated with the density-corrected and self-consistent functionals, respectively.

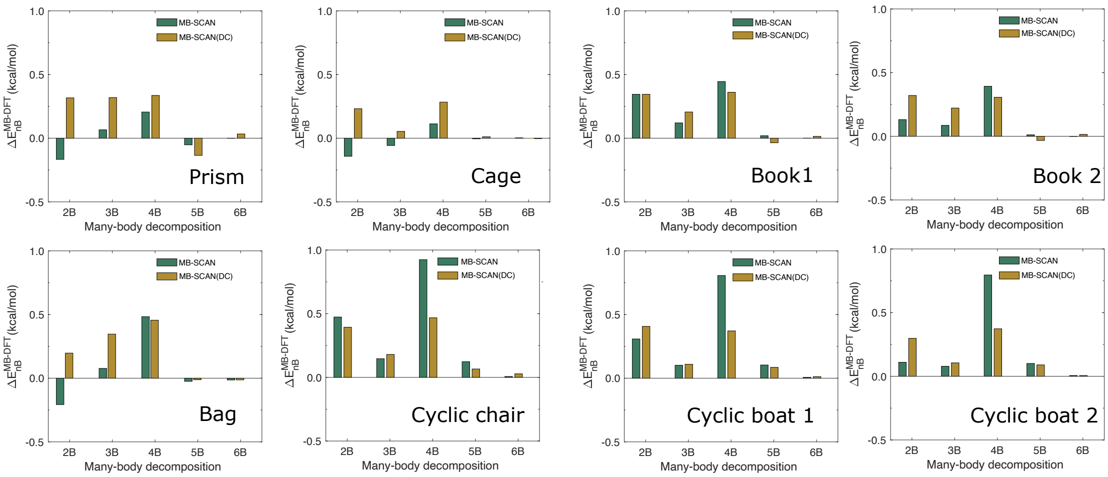

Supplementary Figure 5: **Differences between MB-DFT and DFT many-body energies.** Errors in nB energies (with $n = 2 - 6$) associated with the MB-SCAN and MB-SCAN(DC) PEFs relative to the values calculated with corresponding parent SCAN and DC-SCAN functionals for the first eight low-energy isomers of the water hexamer. Errors are defined as $\Delta E^{n\mathrm{B}}_{\mathrm{MB-DFT}} = E^{n\mathrm{B}}_{\mathrm{MB-DFT}} - E^{n\mathrm{B}}_{\mathrm{DFT}}$, where $E^{n\mathrm{B}}_{\mathrm{MB-DFT}}$ and $E^{n\mathrm{B}}_{\mathrm{DFT}}$ are the nB energies calculated with the MB-DFT PEFs and corrresponding DFT functionals, respectively.

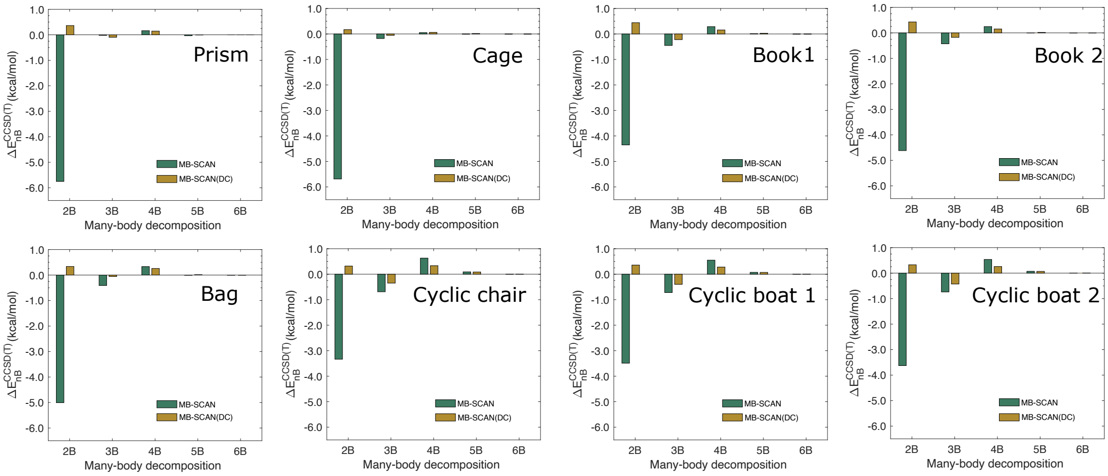

Supplementary Figure 6: **Differences between MB-DFT and CCSD(T)/CBS many-body energies.** Errors in nB energies (with $n = 2 - 6$) associated with the MB-SCAN and MB-SCAN(DC) PEFs relative to the corresponding CCSD(T)/CBS reference values of ref. 1 calculated for the first eight low-energy isomers of the water hexamer. Errors are defined as $\Delta E^{n\mathrm{B}}_{\mathrm{CCSD(T)}} = E^{n\mathrm{B}}_{\mathrm{MB-DFT}} - E^{n\mathrm{B}}_{\mathrm{CCSD(T)}}$, where $E^{n\mathrm{B}}_{\mathrm{MB-DFT}}$ and $E^{n\mathrm{B}}_{\mathrm{CCSD(T)}}$ are the nB energies calculated with the MB-DFT PEFs and at the CCSD(T)/CBS level of theory, respectively.

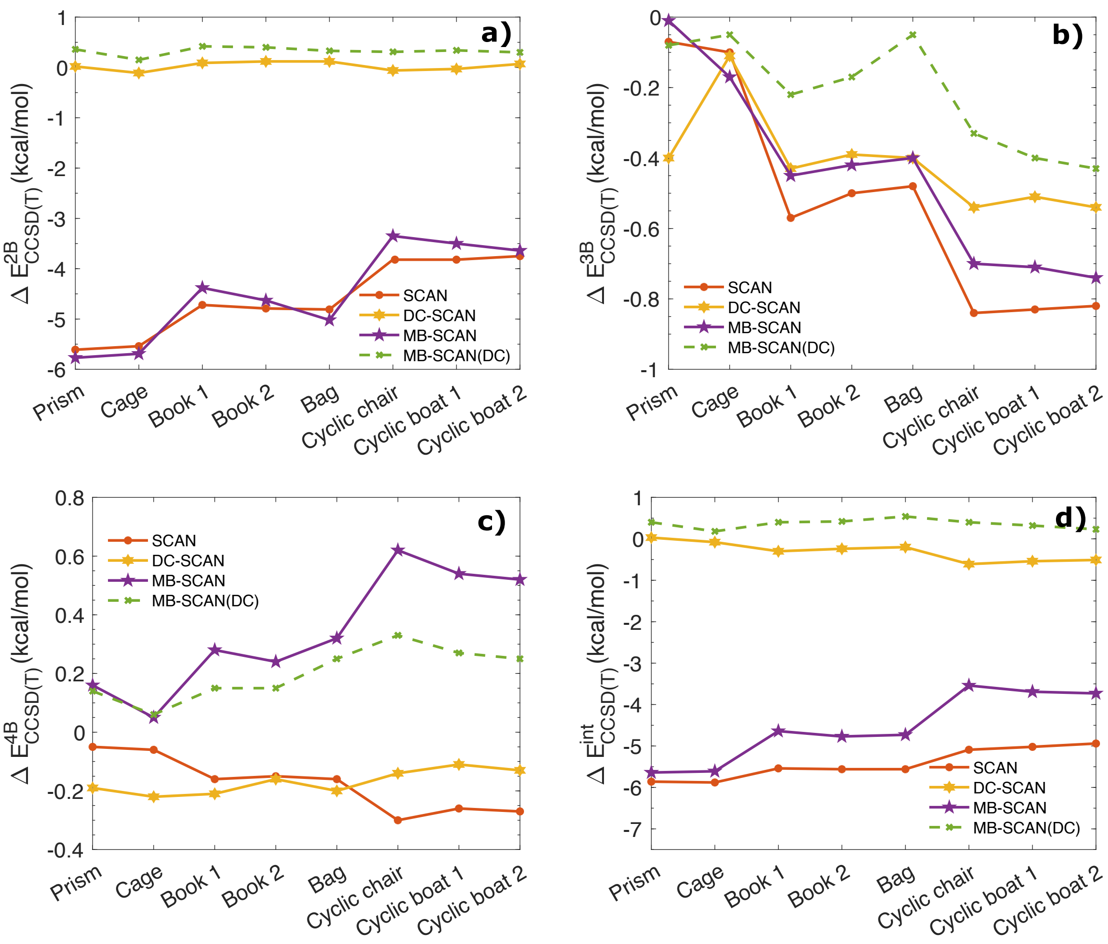

Supplementary Figure 7: **Errors of DFT and MB-DFT interaction energies components.** Errors in the nB energies (with $n = 2 - 4$) and interaction energies associated with the SCAN and DC-SCAN functionals, and corresponding MB-SCAN and MB-SCAN(DC) PEFs, relative to the CCSD(T)/CBS reference values of ref. 1 calculated for the first eight low-energy isomers of the water hexamer. Errors are defined as $\Delta E^{n\mathrm{B}}_{\mathrm{CCSD(T)}} = E^{n\mathrm{B}}_{\mathrm{DFT/MB-DFT}} - E^{n\mathrm{B}}_{\mathrm{CCSD(T)}}$, where $E^{n\mathrm{B}}_{\mathrm{DFT/MB-DFT}}$ and $E^{n\mathrm{B}}_{\mathrm{CCSD(T)}}$ are the nB energies calculated with the DFT functionals or MB-DFT PEFs and at the CCSD(T)/CBS level of theory, respectively.

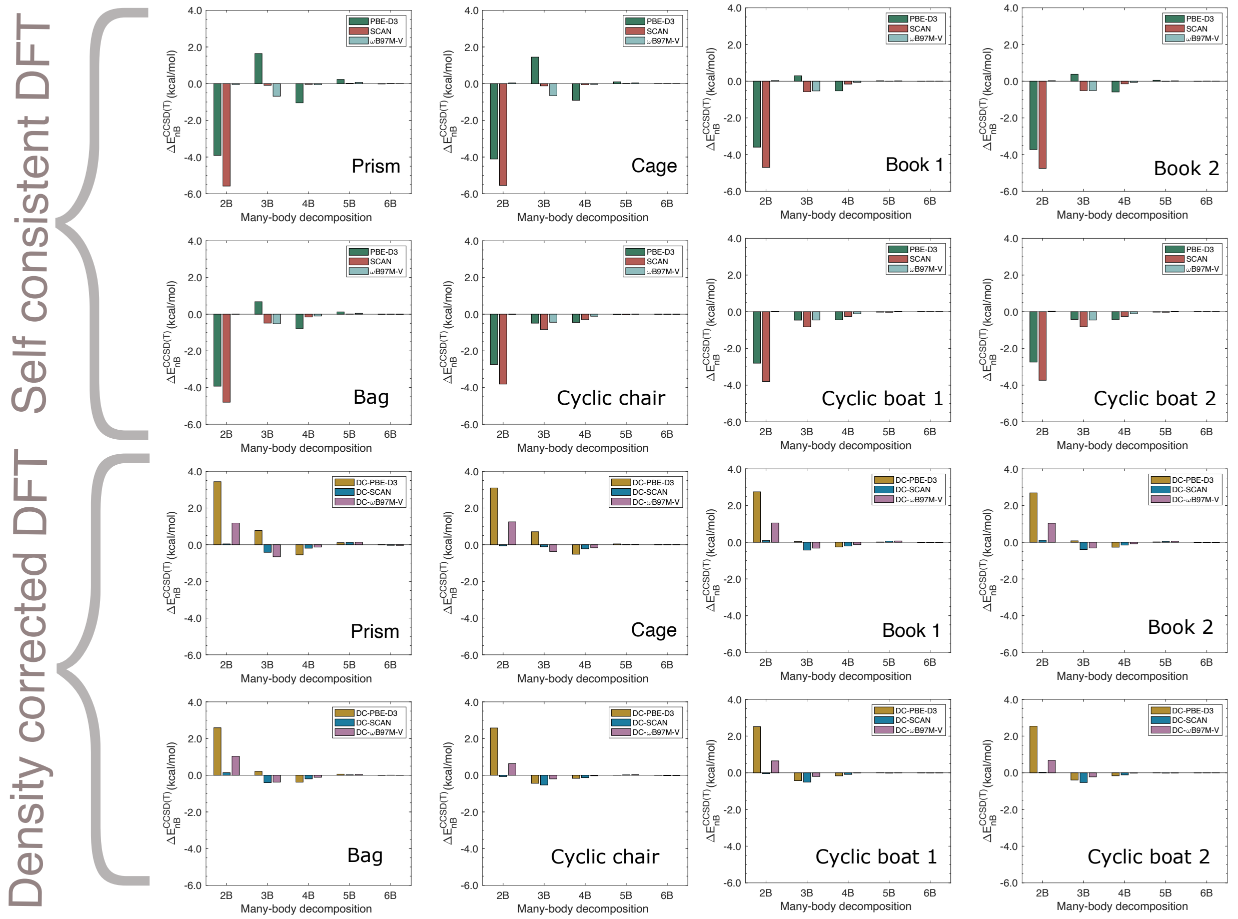

Supplementary Figure 8: **Differences between SC-DFT and DC-DFT many-body energies relative to CCSD(T)/CBS.** Errors in nB energies (with $n = 2 - 6$) associated with the self-consistent PBE-D3, SCAN and ωB97M-V functionals and the corresponding density-corrected DC-PBE-D3, DC-SCAN and DC-ωB97M-V functionals relative to the CCSD(T)/CBS reference values of ref. 1 calculated for the first eight low-energy isomers of the water hexamer. Errors are defined as $\Delta E^{n\mathrm{B}}_{\mathrm{CCSD(T)}} = E^{n\mathrm{B}}_{\mathrm{DC-DFT}} - E^{n\mathrm{B}}_{\mathrm{CCSD(T)}}$, where $E^{n\mathrm{B}}_{\mathrm{DC-DFT}}$ and $E^{n\mathrm{B}}_{\mathrm{CCSD(T)}}$ are the nB energies calculated with the MB-DFT PEFs and at the CCSD(T)/CBS level of theory, respectively.

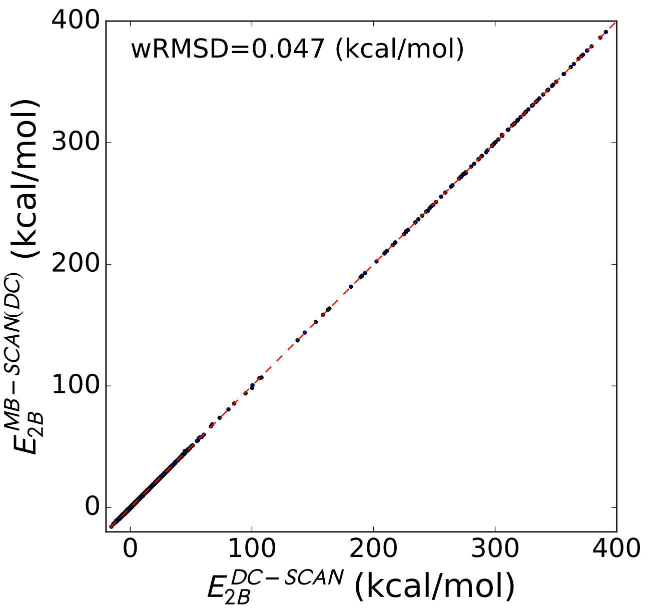

Supplementary Figure 9: **2B energies.** Correlation plot between DC-SCAN (x-axis) and MB-SCAN(DC) (y-axis) 2-body energies. Also shown is the weighted root-mean-square deviation (wRMSD) calculated using the procedure described in ref. 3.

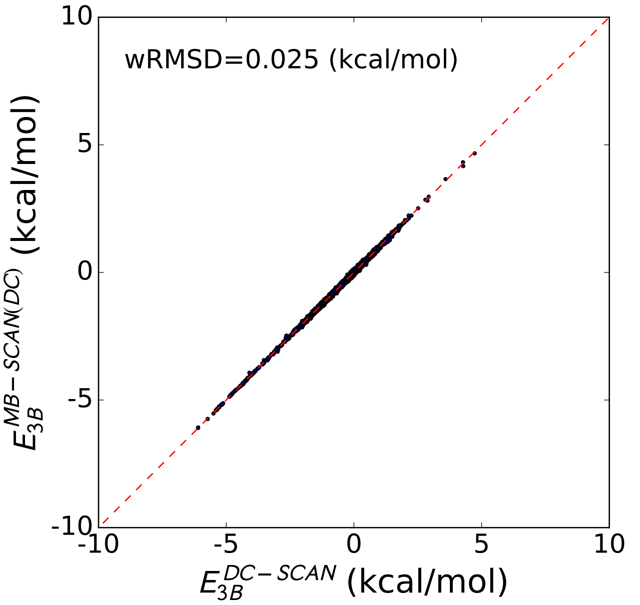

Supplementary Figure 10: **3B energies.** Correlation plot between DC-SCAN (x-axis) and MB-SCAN(DC) (y-axis) 3-body energies. Also shown is the weighted root-mean-square deviation (wRMSD) calculated using the procedure described in ref. 4.

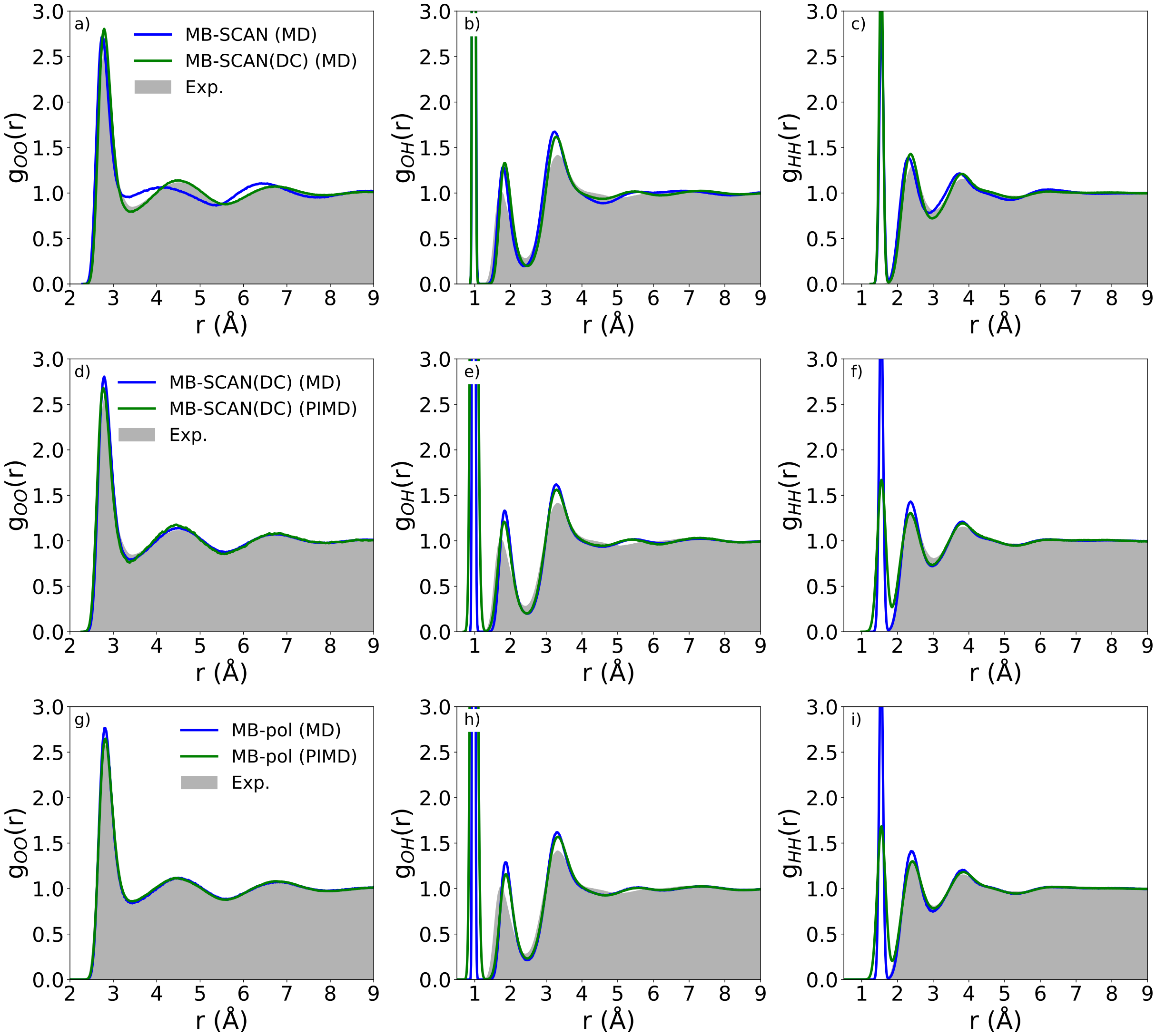

Supplementary Figure 11: **Structure of liquid water.** Oxygen-oxygen (g_{OO}), oxygen-hydrogen (g_{OH}), and hydrogen-hydrogen (g_{HH}) radial distribution functions (RDFs) calculated from NPT simulations carried out. **a-c)** Classical (MD) molecular dynamics simulations with MB-SCAN and MB-SCAN(DC). **d-f)** Classical (MD) and path-integral (PIMD) molecular dynamics simulations with MB-SCAN(DC). **g-i)** Classical (MD) and path-integral (PIMD) molecular dynamics simulations with MB-pol from ref. 5. The experimental O-O RDF is taken from ref. 6, while the experimental O-H and H-H RDFs are taken from ref. 7. All simulations were carried out at 298 K and 1 atm.

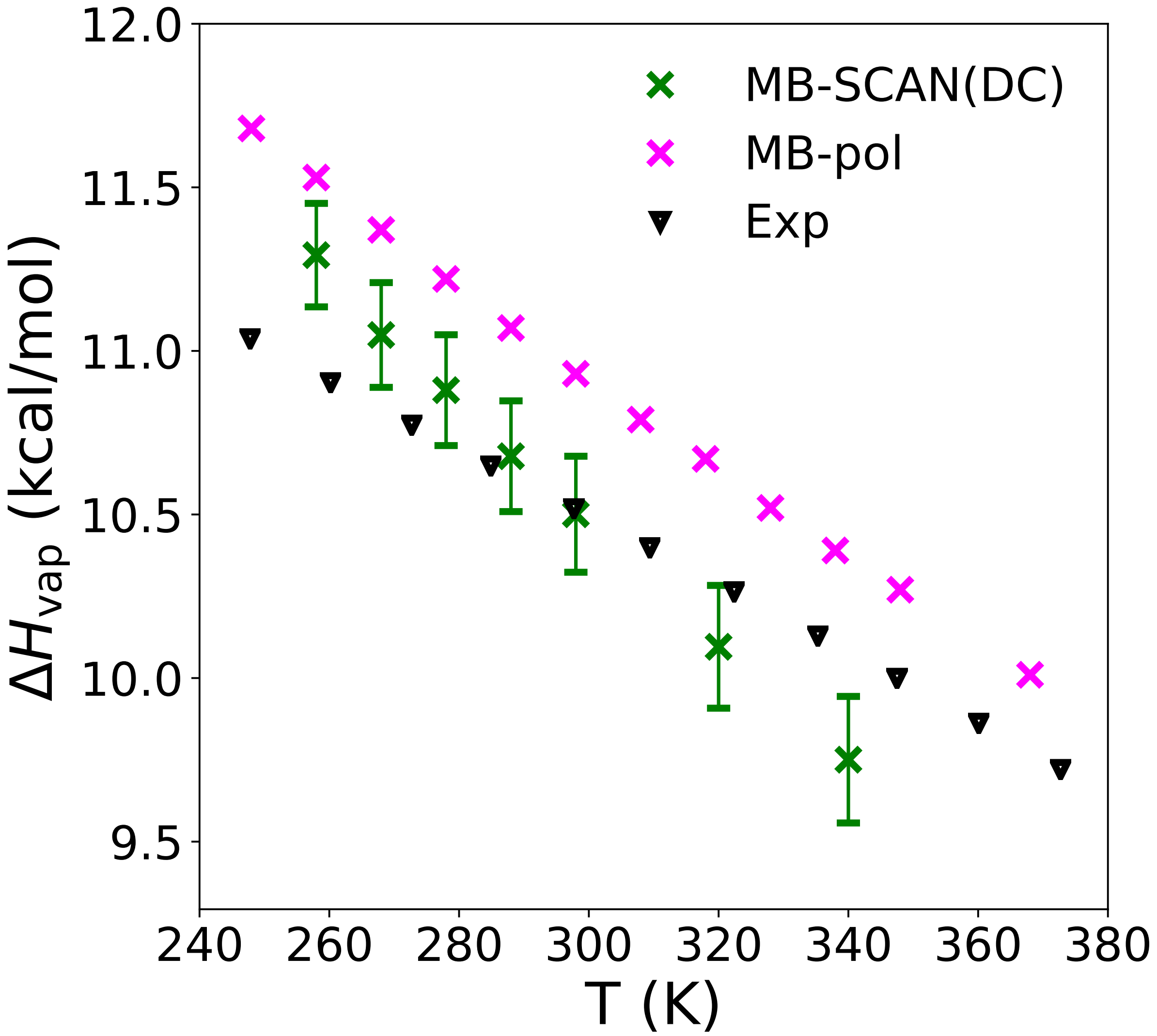

Supplementary Figure 12: **Enthalpy of vaporization of liquid water.** Temperature dependence of the enthalpy of vaporization calculated from classical (MD) simulations of liquid water carried out with the MB-SCAN(DC) PEF in the NPT ensemble as a function of temperature at 1 atm. The MB-pol results are from ref. 1, and the experimental data are from ref. 8. As discussed in ref. 1, the enthalpy of vaporization is significantly affected by nuclear quantum effects, which are neglected in the classical MD simulations presented here, especially at lower temperatures.

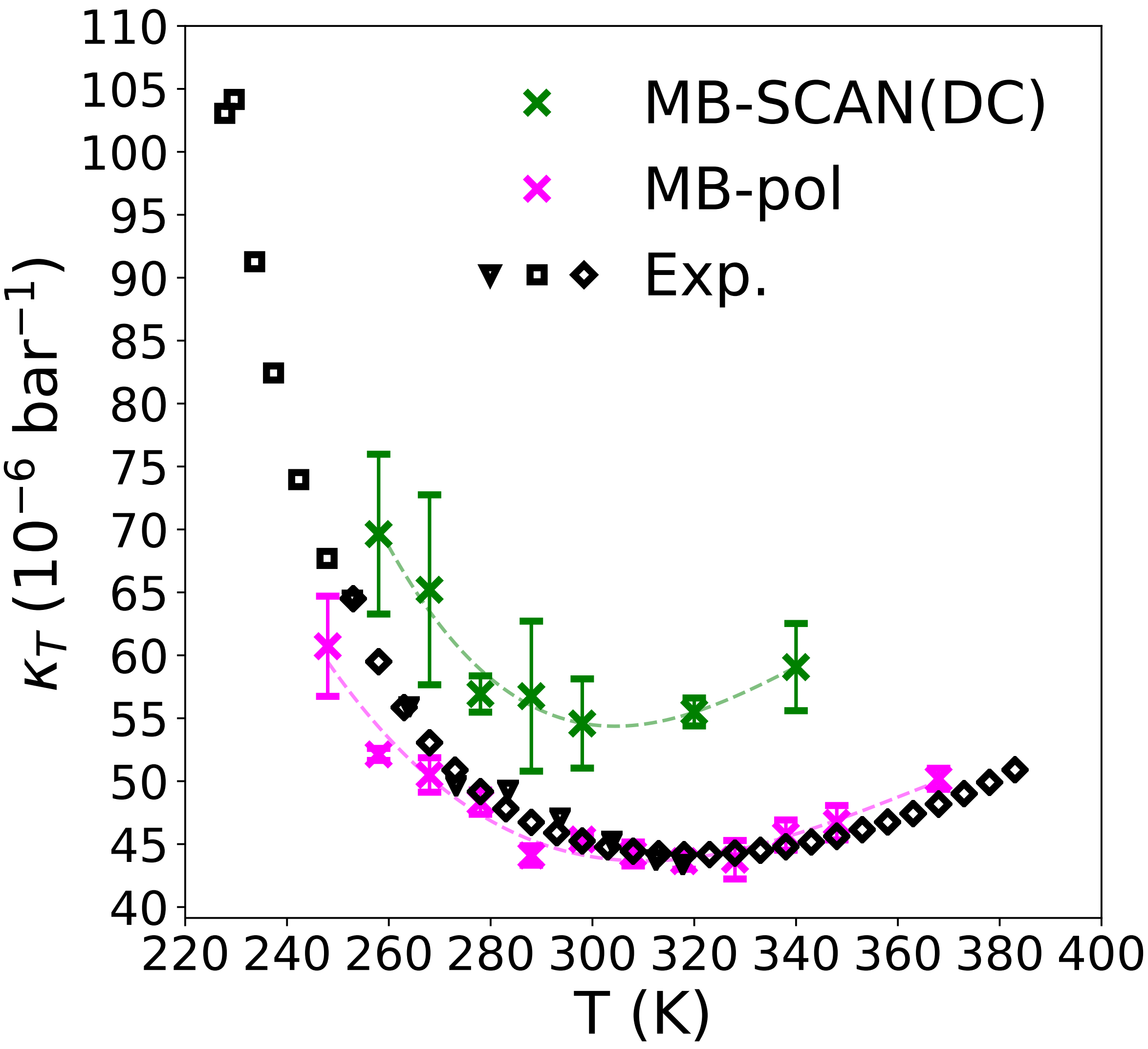

Supplementary Figure 13: **Isothermal compressibility of liquid water.** Temperature dependence of the isothermal compressibility calculated from classical (MD) simulations of liquid water carried out with the MB-SCAN(DC) PEF in the NPT ensemble as a function of temperature at 1 atm. The MB-pol results are from ref. 1, and the experimental data are from ref. 9 (triangles), ref. 10 (squares), and ref. 11 (diamonds).

Supplementary Tables

Supplementary Table 1: Interaction energies of the water dimer calculated with the SCAN and DC-SCAN functionals for the corresponding optimized geometries using different integration grids.

Grid	SCAN (kcal/mol)	DC-SCAN (kcal/mol)
SG-1	-5.54	-5.02
SG-2	-5.50	-4.97
SG-3	-5.50	-4.98
(99,590)	-5.52	-4.99
(250,974)	-5.51	-4.98

Supplementary Table 2: 2B energies of the first eight low-energy isomers of the water hexamer calculated with SCAN, MB-SCAN, DC-SCAN and MB-SCAN(DC), along with the corresponding CCSD(T)/CBS reference values from ref. 1. All energies are in kcal/mol.

	CCSD(T)/CBS	SCAN	MB-SCAN	DC-SCAN	MB-SCAN(DC)
Prism	-38.94	-44.55	-44.71	-38.92	-38.58
Cage	-38.47	-44.01	-44.16	-38.58	-38.32
Book 1	-36.02	-40.74	-40.40	-35.93	-35.60
Book 2	-36.13	-40.92	-40.76	-36.01	-35.73
Bag	-35.28	-40.09	-40.30	-35.16	-34.95
Cyclic chair	-32.71	-36.53	-36.06	-32.77	-32.40
Cyclic boat 1	-32.30	-36.12	-35.80	-32.33	-31.96
Cyclic boat 2	-32.24	-35.99	-35.88	-32.17	-31.94

Supplementary Table 3: 3B energies of the first eight low-energy isomers of the water hexamer calculated with SCAN, MB-SCAN, DC-SCAN and MB-SCAN(DC), along with the corresponding CCSD(T)/CBS reference values from ref. 1. All energies are in kcal/mol.

	CCSD(T)/CBS	SCAN	MB-SCAN	DC-SCAN	MB-SCAN(DC)
Prism	-8.70	-8.77	-8.71	-9.10	-8.78
Cage	-8.97	-9.07	-9.14	-9.08	-9.02
Book 1	-10.38	-10.95	-10.83	-10.81	-10.60
Book 2	-10.11	-10.61	-10.53	-10.50	-10.28
Bag	-10.35	-10.83	-10.75	-10.75	-10.40
Cyclic chair	-11.78	-12.62	-12.48	-12.32	-12.11
Cyclic boat 1	-11.34	-12.17	-12.05	-11.85	-11.74
Cyclic boat 2	-11.34	-12.16	-12.08	-11.88	-11.77

Supplementary Table 4: 4B energies of the first eight low-energy isomers of the water hexamer calculated with SCAN, MB-SCAN, DC-SCAN and MB-SCAN(DC), along with the corresponding CCSD(T)/CBS reference values from ref. 1. All energies are in kcal/mol.

	CCSD(T)/CBS	SCAN	MB-SCAN	DC-SCAN	MB-SCAN(DC)
Prism	-0.66	-0.71	-0.50	-0.85	-0.52
Cage	-0.53	-0.59	-0.48	-0.75	-0.47
Book 1	-1.08	-1.24	-0.80	-1.29	-0.93
Book 2	-1.00	-1.15	-0.76	-1.16	-0.85
Bag	-1.16	-1.32	-0.84	-1.36	-0.91
Cyclic chair	-1.78	-2.08	-1.16	-1.92	-1.45
Cyclic boat 1	-1.63	-1.89	-1.09	-1.74	-1.36
Cyclic boat 2	-1.61	-1.88	-1.09	-1.74	-1.36

Supplementary Table 5: Interactions energies of the first eight low-energy isomers of the water hexamer calculated with SCAN, MB-SCAN, DC-SCAN and MB-SCAN(DC), along with the corresponding CCSD(T)/CBS reference values from ref. 1. All energies are in kcal/mol.

	CCSD(T)/CBS	SCAN	MB-SCAN	DC-SCAN	MB-SCAN(DC)
Prism	-48.24	-54.10	-53.88	-48.21	-47.84
Cage	-47.95	-53.83	-53.56	-48.03	-47.77
Book 1	-47.52	-53.06	-52.16	-47.82	-47.12
Book 2	-47.26	-52.82	-52.03	-47.50	-46.84
Bag	-46.80	-52.36	-51.53	-47.00	-46.26
Cyclic chair	-46.47	-51.56	-50.01	-47.08	-46.07
Cyclic boat 1	-45.44	-50.46	-49.13	-45.98	-45.12
Cyclic boat 2	-45.36	-50.30	-49.09	-45.87	-45.13

Supplementary References

1. Reddy, S. K. *et al.* On the accuracy of the MB-pol many-body potential for water: Interaction energies, vibrational frequencies, and classical thermodynamic and dynamical properties from clusters to liquid water and ice. *J. Chem. Phys.* **145**, 194504 (2016).

2. Brandenburg, J., Bates, J., Sun, J. & Perdew, J. Benchmark tests of a strongly constrained semilocal functional with a long-range dispersion correction. *Phys. Rev. B* **94**, 115144 (2016).

3. Babin, V., Leforestier, C. & Paesani, F. Development of a "first principles" water potential with flexible monomers: Dimer potential energy surface, VRT spectrum, and second virial coefficient. *J. Chem. Theory Comput.* **9**, 5395–5403 (2013).

4. Babin, V., Medders, G. R. & Paesani, F. Development of a "first principles" water potential with flexible monomers. II: Trimer potential energy surface, third virial coefficient, and small clusters. *J. Chem. Theory Comput.* **10**, 1599–1607 (2014).

5. Medders, G. R., Babin, V. & Paesani, F. Development of a "first-principles" water potential with flexible monomers. III. Liquid phase properties. *J. Chem. Theory Comput.* **10**, 2906–2910 (2014).

6. Skinner, L. B. *et al.* Benchmark oxygen-oxygen pair-distribution function of ambient water from X-ray diffraction measurements with a wide Q-range. *J. Chem. Phys.* **138**, 074506 (2013).

7. Soper, A. & Benmore, C. Quantum differences between heavy and light water. *Phys. Rev. Lett.* **101**, 065502 (2008).

8. Wagner, W. & Pruß, A. The iapws formulation 1995 for the thermodynamic properties of ordinary water substance for general and scientific use. *J. Phys. Chem. Ref. Data* **31**, 387–535 (2002).

9. Speedy, R. & Angell, C. Isothermal compressibility of supercooled water and evidence for a thermodynamic singularity at -45 °c. *J. Chem. Phys.* **65**, 851–858 (1976).

10. Kim, K. H. *et al.* Maxima in the thermodynamic response and correlation functions of deeply supercooled water. *Science* **358**, 1589–1593 (2017).

11. Kell, G. S. Isothermal compressibility of liquid water at 1 atm. *J. Chem. Eng. Data* **15**, 119–122 (1970).
